# Correlation of Serum *β*-Endorphin and the Quality of Life in Allergic Rhinitis

**DOI:** 10.1155/2016/2025418

**Published:** 2016-08-25

**Authors:** Jichao Sha, Cuida Meng, Lin Li, Na Cui, Qian Xiu, Dongdong Zhu

**Affiliations:** Department of Otorhinolaryngology, Head and Neck Surgery, China-Japan Union Hospital of Jilin University, 126 Xiantai Blvd, Changchun 130033, China

## Abstract

*Background.* Allergic rhinitis (AR) significantly impairs the quality of life of the patients; however, a questionnaire alone is an insufficient and subjective measure of this condition. Obtaining an objective clinical assessment of the level of impairment will be valuable for its treatment. *β*-Endorphin is one of the most important mediators of both mental state and specific immunity. Thus, we investigated the possibility of using *β*-endorphin as a biomarker for evaluating the impairment level in AR.* Methods.* This study included 48 patients with AR and 32 healthy volunteers. The serum *β*-endorphin level was determined by enzyme immunoassay, and the serum-specific IgE and total IgE levels were determined by immunoblot assay. The Rhinoconjunctivitis Quality of Life Questionnaire (RQLQ) was used to assess the impairment level in the symptom duration.* Results.* The *β*-endorphin concentration was significantly decreased in AR patients compared to the healthy controls (*p* = 0.000, *p* < 0.05). There was significant negative correlation between the impairment level and serum *β*-endorphin level (correlation coefficient: −0.468; *p* = 0.001; *p* < 0.05), but there was no association between the serum *β*-endorphin and total IgE levels (*p* = 0.947, *p* > 0.05).* Conclusion. β*-Endorphin is a systemic biomarker that has the potential to assess the impairment level in AR and may therefore be a novel therapeutic target for the treatment of AR.

## 1. Introduction

Allergic rhinitis (AR) is a symptomatic disorder involving the nose and eyes and is caused by exposure to allergens. It is characterized by sneezing; itching; nasal congestion; rhinorrhea; and generalized symptoms including fatigue, mood changes, depression, anxiety, and impaired ability to work or study. These bothersome symptoms often impair the quality of life (QOL) of the patients [1]. The QOL in patients with AR may be defined as the patient's subjective perception of the impact of AR and the effect on their daily life, physical, psychological, and social function and well-being. Currently, the Rhinoconjunctivitis Quality of Life Questionnaire (RQLQ) is the most widely used questionnaire in AR patients [2]. Reducing the impairment degree is one of the therapeutic goals. In addition to subjective measures such as the RQLQ, a more precise measure of symptoms could help clinicians to determine the severity of the condition and accordingly identify the correct treatment approach for the symptoms.

The immunopathology and psychological state of AR are the main factors affecting the QOL in patients with AR. The opioid peptide *β*-endorphin is one of the most important mediators of both the immune response [3, 4] and mental state [5, 6]. Data gathered over the past decades indicate that *β*-endorphin is an important peptide for maintaining the homeostasis of behavioral, cognitive, and neuroendocrine functions and mediating the Th1-Th2-type response switch [7]. Thus, we hypothesized that *β*-endorphin has potential as a biomarker for evaluating the QOL in AR patients. In this study, we investigated the serum *β*-endorphin level in patients with AR and in healthy controls and evaluated the associations between the serum *β*-endorphin level and RQLQ scores as well as total IgE grade in the symptomatic stage in AR patients.

## 2. Materials and Methods

### 2.1. Study Participants

Participants were recruited from the Clinic of Otorhinolaryngology, Head and Neck Surgery of our hospital. Patients with AR were selected according to the diagnostic criteria proposed by ARIA (symptoms, positive skin prick test, and positive serum-specific IgE) and were in the age range of 18–65 years [8]. The patients had not received any treatment for at least 1 month prior to the study. None of the recruited participants had any other immunological diseases or infection for 2 months prior to the study; those with hypertension, psychiatric disease, and substance abuse were excluded from the study. As certain conditions such as asthma, opioid medications, and smoking may affect plasma beta-endorphin levels, study participants with a history of asthma, those who were smokers, and those who were taking opioid medications were excluded from the study.

Thirty-two healthy volunteers (14 men and 18 women; mean ± SD age: 34.8 ± 10.7) and forty-eight patients with AR (22 men and 26 women; mean ± SD age: 33.4 ± 10.5) were recruited for participation in this study. Serum samples were collected from all patients and healthy volunteers between 8 AM and 10 AM and stored at −80°C within 1 h until the assay for IgE and *β*-endorphin levels. One patient did not complete the procedure. The study was approved by the Ethics Committee of our Hospital (IRB: 2014ks033), and all the participants gave their informed consent.

### 2.2. Assessment of QOL Impairment Level for Patients with AR

The QOL impairment level was assessed using the RQLQ. The RQLQ contains 28 items in 7 domains, including activity, sleep, nose symptoms, eye symptoms, non-nose/eye symptoms, practical problems, and emotional state. Each item was scored from 0 (not troubling) to 6 (extremely troubling) [9]. The total score for each patient was recorded and interpreted as mild impairment (scores 0–56), moderate impairment (scores 57–112), or severe impairment (scores 113–168).

### 2.3. Assays of Serum *β*-Endorphin and IgE Levels

The concentration of *β*-endorphin (pg/mL) in the serum was assayed using a commercially available ELISA Kit (Cat: CSB-E06821h, Wuhan Huamei Biotech Co., Ltd., China) according to the manufacturer's instructions. The total IgE was detected using an AllergyScreen Test Kit (LOT: K-150616, Mediwiss Analytic GmbH, Germany), which indicates if the total IgE content is less than 100 KU/L, between 100 and 200 KU/L, or higher than 200 KU/L.

### 2.4. Statistical Methods

Data were analyzed using SPSS 17.0 (SPSS, Inc., Chicago, IL). An unpaired *t*-test was used to compare the *β*-endorphin concentrations between the AR and healthy control subjects. One-way analysis of variance (ANOVA) for the three groups and Spearman's rank correlation coefficient (as continuous variable) were used to assess the relationship between the serum *β*-endorphin level, the RQLQ score, and the total IgE grade. A two-tailed value of *p* < 0.05 indicated statistical significance.

## 3. Results

**Figure 1:**
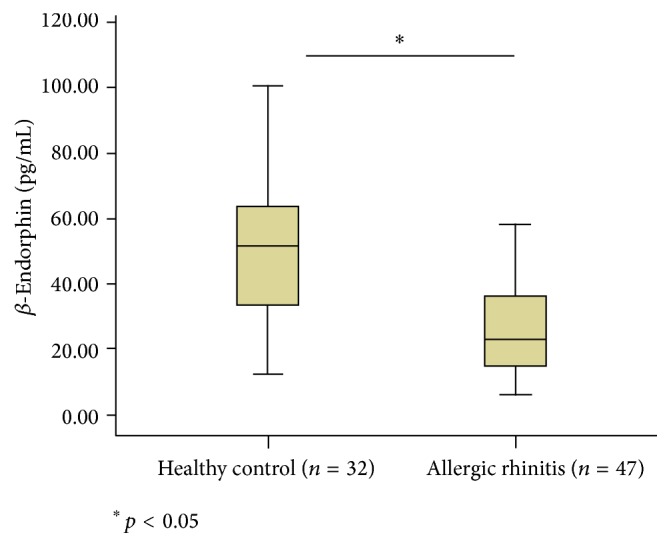
Serum *β*-endorphin level was significantly (*p* = 0.000, *p* < 0.05) reduced in patients with allergic rhinitis compared to healthy controls.

The serum *β*-endorphin level was significantly (*p* = 0.000, *p* < 0.05) reduced in the AR patient group (25.93 ± 13.87 pg/mL) compared to healthy controls (50.424 ± 20.729 pg/mL) (Figure 1).

The mean ± SD RQLQ score of AR patients was 67.96 ± 31.74. The severity of impairment was classified into three categories: mild (scores 0–56), moderate (scores 57–112), and severe (scores 113–168). In the mild group, the mean score was 38.47 ± 11.93 (*n* = 19). In the moderate group, the mean score was 79.70 ± 16.19 (*n* = 23). In the severe group, the mean score was 126 ± 19.18 (*n* = 5). The mean *β*-endorphin concentrations in the mild, moderate, and severe groups were 33.65 ± 15.73, 22.27 ± 9.59, and 11.64 ± 2.64 pg/mL, respectively (Table 1). The serum *β*-endorphin level and RQLQ scores were negatively correlated between both the classified groups (ANOVA, *F* = 8.178, *p* = 0.001) and the scores (correlation coefficient: −0.468, *p* = 0.001). However, there were no correlations between the *β*-endorphin concentration and total IgE grade (*p* = 0.947, >0.05; Table 2).

## 4. Discussion

AR historically exhibits a level of impairment on the QOL of patients, and the major aim of treatment is to improve the QOL of patients. Thus, the assessment of AR is extremely important in terms of both diagnosis and therapeutic effects. However, the current assessment approach represents a patient's perception of the state of health using a questionnaire [10], which is perceptual and not physiological. There is a need for additional measures to verify the clinical relevance of the degree of impairment on QOL. RQLQ is the most frequently used measure at present, and allergic symptoms and emotional function are two factors that contribute to worsening or improvement of the QOL [11]. The role of *β*-endorphin has been the subject of various studies in allergic diseases and mental health problems in recent years, although very little has been published about the role of this neurohormone in AR.

The opioid *β*-endorphin, a 31-amino-acid-long pro-opiomelanocortin (POMC) molecule, is associated with the activity of the stress-sensitive hypothalamic-pituitary-adrenal (HPA) axis [12, 13]. The inhibitory property of *β*-endorphin is widely known in the regulation of immune function [14]. Reduced *β*-endorphin levels have been reported in human rheumatoid arthritis, osteoarthritis, systemic lupus erythematosus, gout, ankylosing spondylitis, pseudogout, and psoriatic arthritis [15] as well as in autoimmune diseases in animal models [16]. In our study, the results show that the serum *β*-endorphin level was significantly decreased in patients with AR compared to healthy controls. Results from a previous colorectal hypersensitivity study showed that mice that developed hypersensitivity have reduced expression of the *β*-endorphin gene and suggest that *β*-endorphin acts as a peripheral mediator in hypersensitivity [17]. Reports by Lee et al. on atopic dermatitis demonstrated that *β*-endorphin is an independent biological marker for allergic symptoms and disease severity [18–20]. In their studies, they measured the serum IgE and found that it is a marker in atopic dermatitis; however, in our study, we found no relationship between serum IgE and AR severity. A pediatric study also observed a reduction in the serum *β*-endorphin level in patients with atopic dermatitis [21]. Investigations into lower airway allergic diseases, such as asthma and chronic obstructive pulmonary disease, have demonstrated that the *β*-endorphin response has been shown to decrease the activation of respiratory muscles and change the pattern of breathing to a more rapid and shallow one [22]. These studies suggest that *β*-endorphin may play a crucial role in allergic diseases such as AR, which is similar to the findings of our study. Another study found a statistically significant reduction in the *β*-endorphin concentrations after histamine provocation in patients with asthma [23].

One study found that *β*-endorphin induces an increase in nasal congestion through the direct neuroendocrine receptor of mast cells that enhances the mediator response to nasal allergen challenge by intranasal *β*-endorphin challenge [24]. In an immunotherapy assessment, a study used the serum *β*-endorphin level as a biomarker and found its statistical significance; however, this study only measured olive and grass pollen immunotherapy in children [25].

These studies focused on the suppression of immunological function of *β*-endorphin by neuroendocrinology mechanisms, including interaction with sensory nerves [26], mast cells [27], blood T lymphocytes [28], and even memory T cells [29]. It is known that the immunopathology of AR and psychological effects are the main factors affecting the QOL in patients. It is noteworthy that the opioid peptide *β*-endorphin is not only a mediator of immune responses but also a predictor of mental health problems [30–32]. Mental health problems such as stress, depressive episodes, and anxiety disorders have been reported to be much more prevalent among patients with AR than among individuals without prevalent AR [33–35]. Although *β*-endorphin has received considerable attention for both its immunological function in allergic disease and its potential to predict the mental state of patients, few studies have investigated the relationship between *β*-endorphin and QOL, which are associated with these two conditions. These previous studies provided evidence that *β*-endorphin is associated with impaired QOL in AR, and our study further explored this possibility. Our results revealed that serum *β*-endorphin and RQLQ scores were highly negatively correlated between both the classified groups and the scores.

This study has some limitations. First, the sample size of our study is small. Second, other covariates such as age, gender, and psychological factors of the participants who filled out the RQLQ were not accounted for. Addressing these limitations in future studies will help verify the findings of the present study.

In conclusion, our study revealed two important findings. First, the serum *β*-endorphin level was obviously reduced in the patients with AR compared to the healthy controls. Second, the *β*-endorphin level was significantly related to the RQLQ severity of the AR.

## Figures and Tables

**Table 1:** Comparison of the *β*-endorphin concentration and RQLQ scores among the three groups according to RQLQ.

	*β*-Endorphin (mean ± SD, pg/mL)	RQLQ scores (mean ± SD)
Mild group (*n* = 19) (scores 0–56)	33.65 ± 15.73	38.47 ± 11.93
Moderate group (*n* = 23) (scores 57–112)	22.27 ± 9.59	79.70 ± 16.19
Severe group (*n* = 5) (scores 113–168)	11.64 ± 2.64	126 ± 19.18

*F* = 8.178	*p* = 0.001^*∗*^	*p* < 0.05^*∗*^
Correlation coefficient: −0.468	*p* = 0.001^*∗*^	*p* < 0.05^*∗*^

Data of each group were compared by single-factor ANOVA.

Correlation analysis was performed using Spearman's correlation test (two-tailed).

^*∗*^ *p* < 0.05.

**Table 2:** Comparison of the *β*-endorphin concentrations and RQLQ scores among the three groups according to total IgE.

	*β*-Endorphin (mean ± SD, pg/mL)	Total IgE
*N* = 13	27.22 ± 17.675	Less than 100 KU/L
*N* = 14	26.34 ± 14.46	100–200 KU/L
*N* = 20	24.67 ± 11.06	More than 200 KU/L

*F* = 0.494	*p* = 0.940	*p* > 0.05

Data between groups were compared by single-factor ANOVA.
